# Primary Mucoepidermoid Carcinoma of the Lung Coexisting With Pulmonary Tuberculosis: A Rare Case Report

**DOI:** 10.7759/cureus.44978

**Published:** 2023-09-10

**Authors:** Pretty Singh, Abhishek Verma, Kavita Somani, Sujatha Poduval, Om Prakash Verma

**Affiliations:** 1 Department of Pathology and Laboratory Medicine, Apollomedics Super Speciality Hospital, Lucknow, IND; 2 Department of Pulmonary Medicine, Apollomedics Super Speciality Hospital, Lucknow, IND

**Keywords:** broncho alveolar lavage (bal), tree-in-bud, immunohistochemistry and biopsy, fiberoptic flexible bronchoscopy, lung neoplasm, salivary gland neoplasm, pulmonar tuberculosis, mucoepidermoid carcinoma (mec)

## Abstract

Mucoepidermoid carcinoma (MEC) is a well-established neoplasm of the salivary glands. However, the MEC of the lung is an exceedingly rare neoplasm that falls under the category of salivary gland-type tumors of the lung. Pulmonary MEC is recognized for its indolent progression. Pulmonary tuberculosis (TB) is a prevalent infectious disease in India and ranks among the leading causes of death from infectious diseases. Nevertheless, the co-occurrence of pulmonary MEC with pulmonary TB is a rare phenomenon that has not been documented in the literature. In this report, we describe a 54-year-old male patient who presented with symptoms of dysphagia, weight loss, and fever. Histopathological examination diagnosed him with pulmonary MEC, and concurrent cytology and Gene-Xpert tests confirmed tuberculosis. This case represents the first documented instance of this particular co-occurrence. It underscores the limitations of radiology in diagnosing such a rare neoplasm, especially when there is an absence of lung parenchyma infiltration and a mass lesion. Additionally, this case supports the possibility of an interdependent relationship between malignancies and tuberculosis.

## Introduction

Primary pulmonary mucoepidermoid carcinoma (MEC) is a rare malignant neoplasm, ranging from 0.1 to 0.2% of the total pulmonary malignancies [1]. It is primarily a salivary gland neoplasm arising from parotid and submandibular glands. However, cases of MEC have been reported in the lung, arising from the tracheobronchial mucus glands. It has a wide age range from 3 to 79 years [2]. Similar to salivary gland neoplasms, primary lung malignancies are also categorized into low, intermediate, and high grades based on the grading system established by the Armed Forces Institute of Pathology (AFIP) for MEC in the salivary glands. Histologically, MEC is composed of a combination of intermediate, squamoid, and mucus cells. High-grade MEC is made up of sheets of squamous and intermediate cells with a lower proportion of mucus cells. In contrast, low-grade MEC contains varying proportions of glandular components, mucus cells, and intermediate cells. A defining feature of a high-grade neoplasm is necrosis [3]. In India, tuberculosis (TB) is a prevalent disease. However, there have been no previous reports of pulmonary TB coexisting with such a rare lung tumor.

## Case presentation

**Figure 1:**
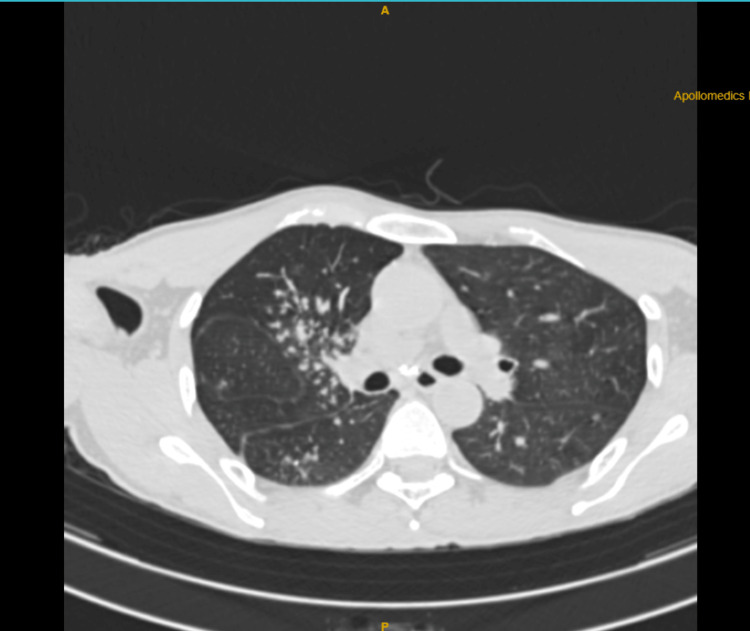
CT of the neck and thorax: tree-in-bud appearance in the right upper lobe of the lung.

A 54-year-old male patient presented to the otorhinolaryngology outpatient department six months ago with chief complaints of hoarseness of voice and dysphagia. On video-laryngoscopy, bilateral adductor cord palsy was noted, and he was advised to have a CT of the neck and thorax along with an MRI of the brain. However, the patient did not return for follow-up. He presented again one month ago with persistent symptoms of dysphagia, hoarseness of voice, and additional complaints of weight loss, loss of appetite, and fever. His CT scan and MRI were conducted. The CT scan of the neck and thorax revealed tracheal luminal narrowing and centrilobular nodules with a tree-in-bud appearance in the right upper lobe of the lung (Figure 1).

**Figure 2:**
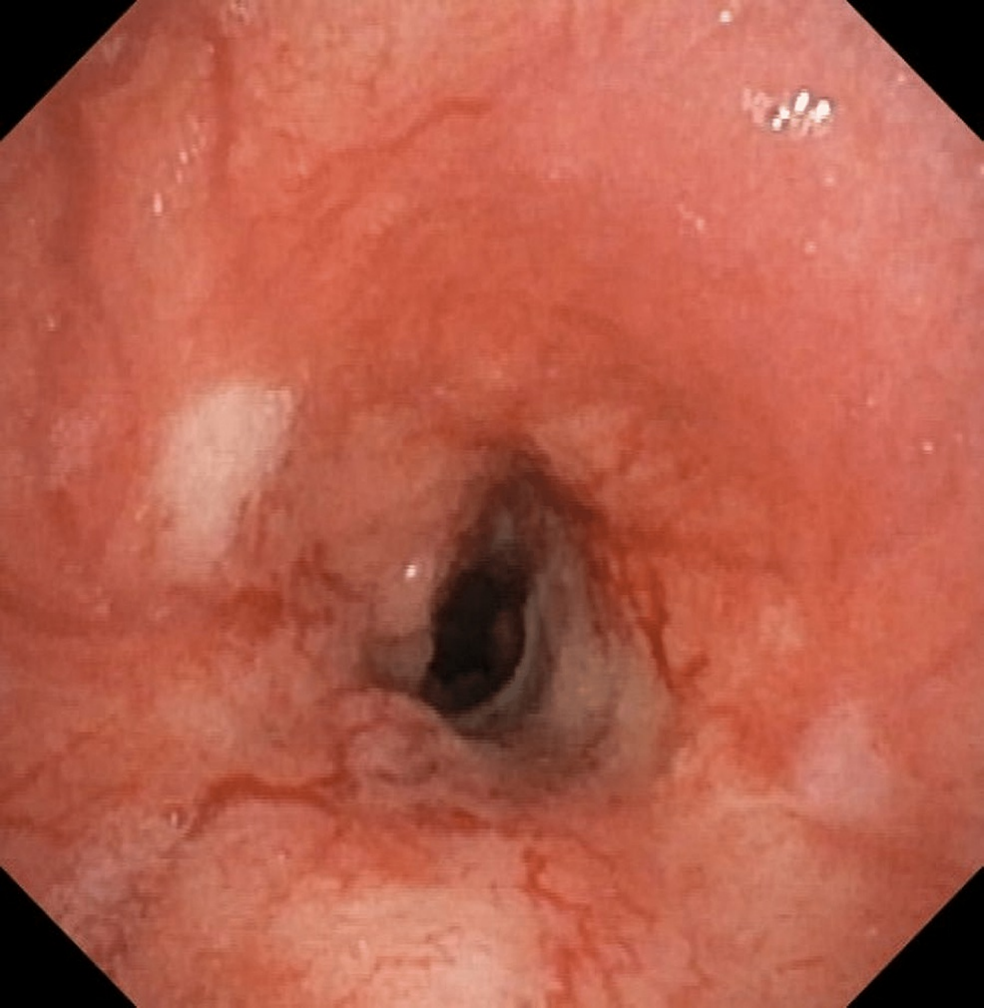
Bronchoscopy: luminal narrowing in the mid-trachea with nodular mucosal infiltrates.

MRI of the brain was suggestive of diffuse cerebral atrophy. The patient was referred to the gastromedicine department, in view of dysphagia, for endoscopy. On endoscopy, the oesophageal lumen showed narrowing due to external compression, with an indentation on the mucosa. However, no luminal growth was observed. A biopsy was taken, which tested negative for malignancy and dysplasia. Upon further workup by the pulmonology department, a bronchoscopy was performed. It revealed significant nodular growth in the mid-trachea, leading to circumferential luminal narrowing (Figure 2).

**Figure 3:**
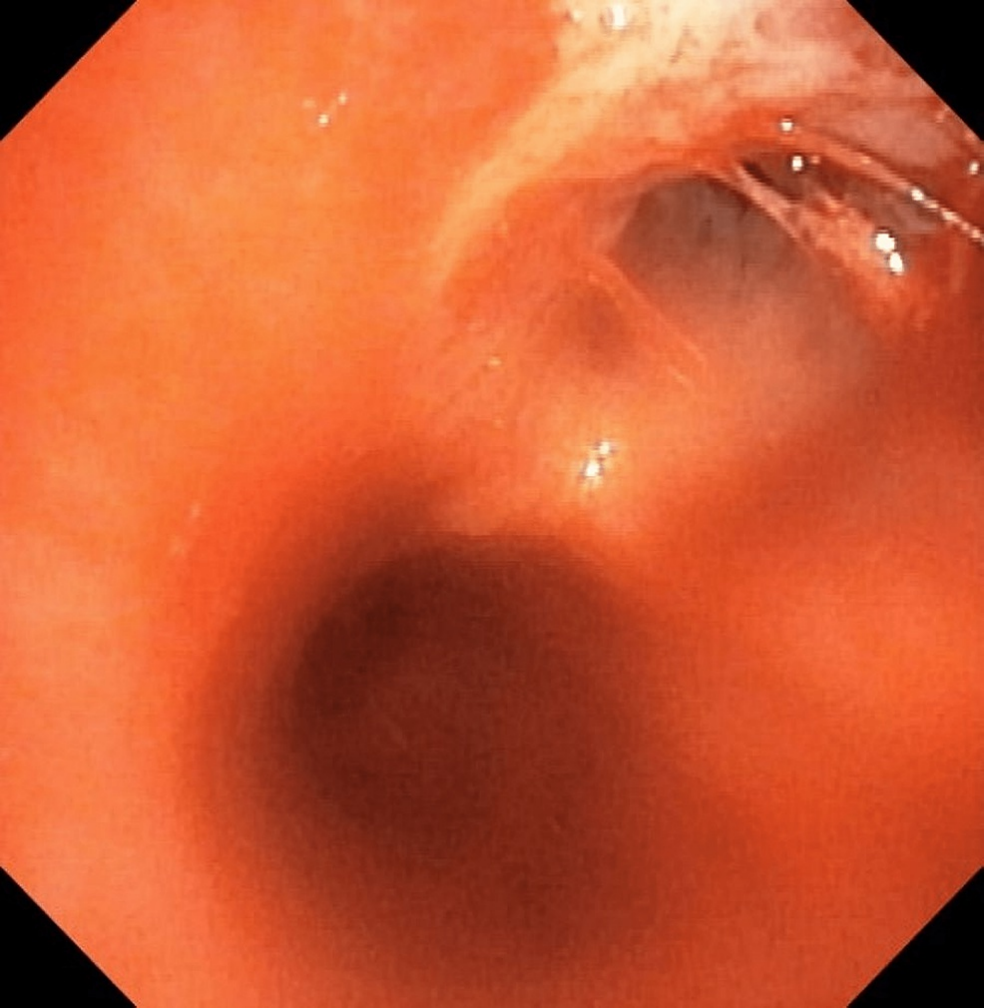
Bronchoscopy: necrotic band in the right upper lobe with mucosal infiltration, suggestive of TB. TB: Tuberculosis.

A biopsy was performed to ascertain the etiology of the nodular growth, with the potential diagnoses being tuberculosis, neoplasm, and amyloidosis. The right upper lobe bronchus displayed necrotic bands (Figure 3). Additionally, a broncho-alveolar lavage (BAL) was obtained from the right upper lobe and sent for both cytology and Gene-Xpert.

**Figure 4:**
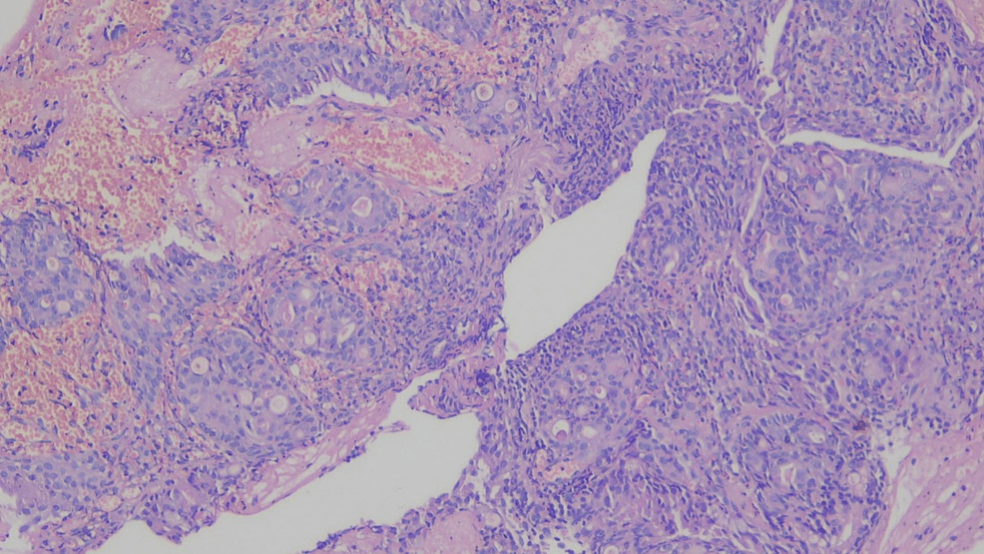
100x magnification: Tumor arranged in a glandular pattern with solid nests of intermediate cells.

**Figure 5:**
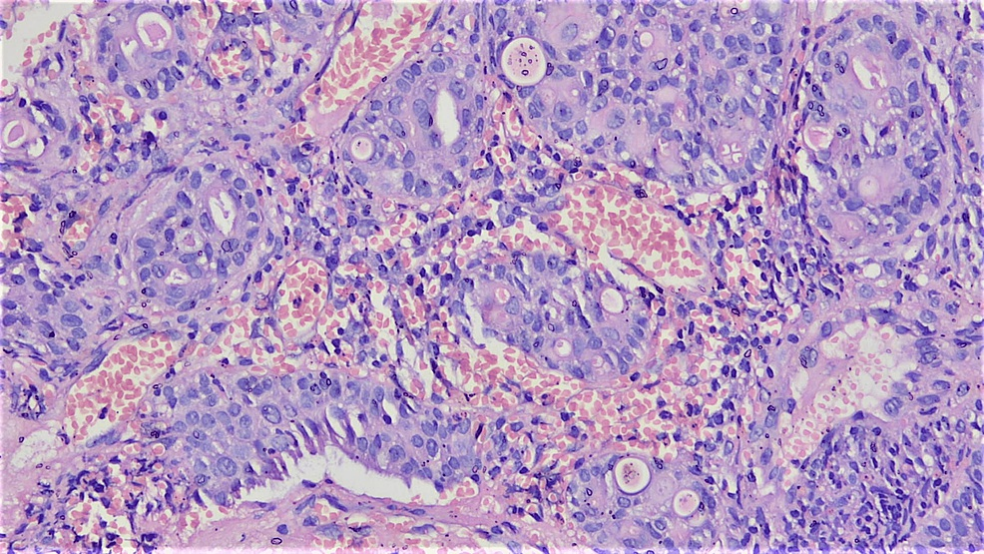
200x magnification: Cribriform pattern with mucus cells and intermediate cells.

On histopathological examination (HPE), the oesophageal biopsy showed strips of hyperplastic and spongiotic stratified squamous epithelium. Mild intraepithelial inflammation was noted. However, there was no evidence of dysplasia or malignancy. On the HPE of the endobronchial biopsy, a malignant epithelial neoplasm was observed. This neoplasm displayed nests and trabeculae of infiltrating tumor cells. However, the overlying respiratory epithelium remained largely unremarkable, with only focal areas of squamous metaplasia. Focal acinar formation with intra-luminal eosinophilic material, as well as cribriforming, was noted. The cells in question were moderately pleomorphic, possessing a high nucleocytoplasmic ratio, fine chromatin, prominent nucleoli, and a moderate amount of amphophilic to vacuolated cytoplasm (suggestive of mucus cells). Focal regions presented nodules of cells with an intermediate appearance (Figures 4-5). Notably, there was no evidence of marked cytological atypia, necrosis, or heightened mitotic activity.

**Figure 6:**
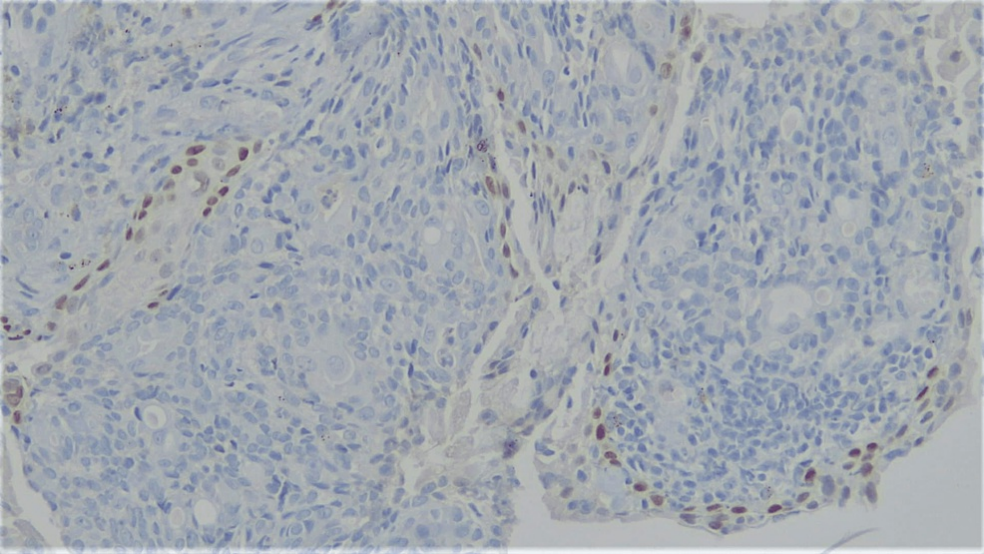
200x magnification, IHC for TTF1: Focal nuclear positivity in the overlying respiratory epithelium, while tumor cells are negative. IHC: Immunohistochemistry.

**Figure 7:**
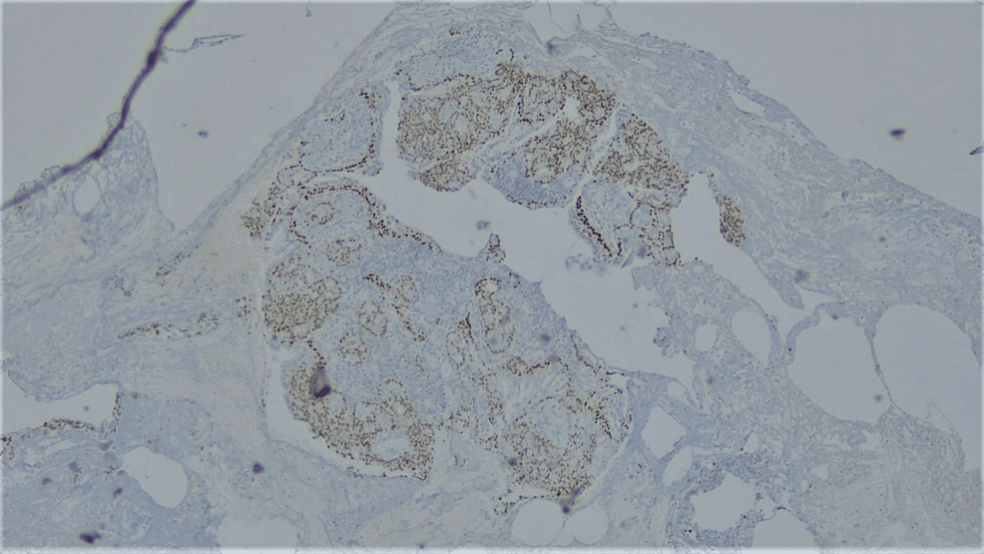
100x magnification, IHC for p40: nuclear positivity in intermediate cells. IHC: Immunohistochemistry.

Histologically, the following differentials were considered: adenoid cystic carcinoma, adenocarcinoma, and MEC. On immunohistochemistry (IHC), TTF1 exhibited focal nuclear positivity in the respiratory epithelium, but it was negative in the tumor cells (Figure 6). Both p40 and p63 showed nuclear positivity in the basal layer of glandular structures as well as in nests of intermediate cells (Figure 7). CK7 was positive in the mucus cells. CD117, Synaptophysin, Chromogranin, CD56, and S100 were all negative in the tumor cells.

**Figure 8:**
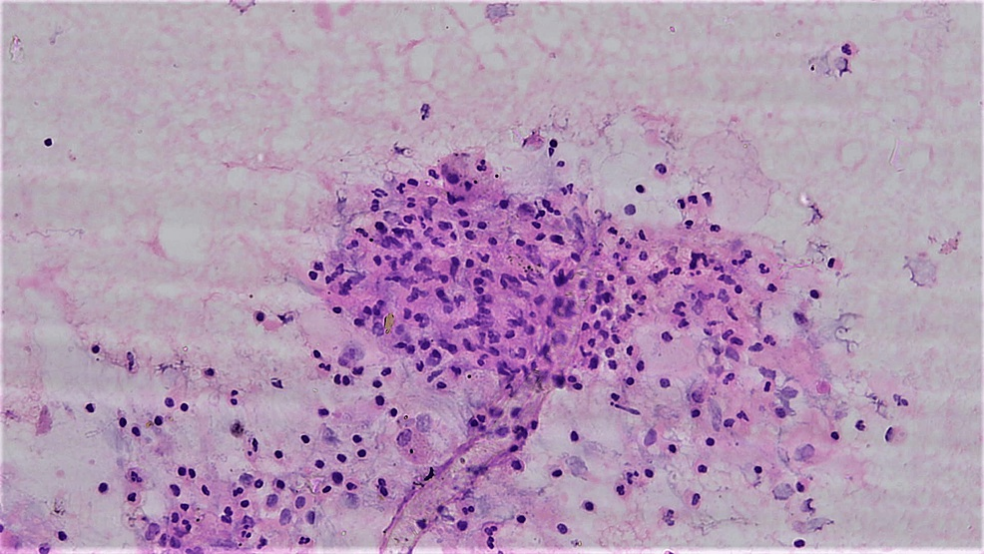
Bronchoalveolar lavage (BAL) cytology at 200x magnification: Neutrophils, lymphocytes, and alveolar macrophages.

**Figure 9:**
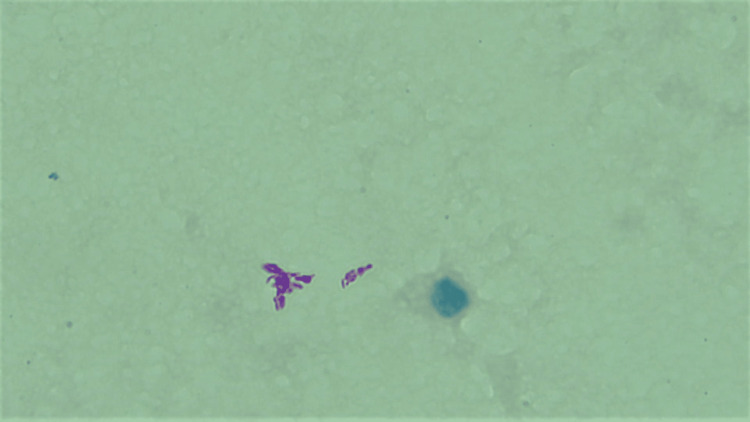
Ziehl-Neelsen stain under oil immersion: Acid-fast bacilli.

Adenocarcinoma was ruled out due to the negativity of TTF1 and Napsin A.Adenoid cystic carcinoma was excluded due to the absence of perineural invasion and the negative immunostaining for CD117 and S100. Based on these findings, a final diagnosis of low-grade MEC (using the AFIP grading system) was rendered. On cytomorphological examination of the BAL, there were a few neutrophils mixed with lymphocytes, alveolar macrophages, and columnar epithelial cells on a mucus-laden dirty background (Figure 8). No evidence of granuloma or malignancy was noted. However, the Ziehl-Neelsen stain revealed a significant number of acid-fast bacilli (Figure 9).

It was reconfirmed by Gene-Xpert MTB, which detected the Mycobacterial Tuberculosis complex. The patient was immediately started on an antitubercular regimen and referred to oncology for further follow-up.

## Discussion

According to the scant data available, primary lung MECs are uncommon tumors with a reported indolent course. A guarded prognosis exists for high-grade MEC, nonetheless. The incidence of TB in India in 2021 was 210 per 100,000 people. According to this data, India is ranked 36th in the world for incidence rates (from largest to smallest incidence numbers). A total of 1.6 million individuals worldwide died from TB in 2021, including 187,000 people with HIV. TB ranks as the second most common infectious killer globally after COVID-19 and stands as the 13th leading cause of death overall. Without treatment, the mortality rate for TB is significant, at about 50% [4]. If either condition is left untreated or undetected, the prognosis for people who have low-grade MEC and TB together could be grim. MEC is primarily found in the peribronchial region. Previous case studies indicate that the tumor has no clear preference for one gender over another [5]. The patient may exhibit various symptoms, such as coughing blood, bronchitis, wheezing, and experiencing chest pain [6]. The co-existence of pulmonary TB in our patient may have caused overlapping symptoms, including fever, weight loss, and loss of appetite. Our patient also presented with an atypical symptom, such as hoarseness of voice. The likely cause of the vocal cord palsy was tracheal involvement, leading to compression of the recurrent laryngeal nerve. However, this was not conclusively established. According to histology, MEC consists of a combination of three distinct cell types in varying ratios: intermediate cells, squamous cells, and mucin-secreting glandular or mucus cells. A key prognostic factor is histological grading, with high-grade MECs showing increased risks for metastasis, recurrence, and mortality [7]. Similar to salivary gland MEC, pulmonary MEC often exhibits the (11;19) translocation, leading to the synthesis of the METC1-MAML2 fusion protein. This protein interferes with the Notch signaling system. This translocation or rearrangement is identifiable through fluorescence in situ hybridization (FISH). While 60-70% of MECs from the salivary glands show this translocation, the exact occurrence percentage in pulmonary MEC is not widely reported due to data and resource limitations [8]. Additionally, according to available data, MEC frequently overexpresses EGFR [9].

Both regional lymph node metastases and underlying lung parenchyma invasion by tumor cells are rare phenomena. Radiology is not helpful and makes it difficult for the doctor to make the diagnosis because there is no mass lesion. The tracheobronchial tree may get narrower, although bronchoscopy can clearly show submucosal nodules. As in this case, the tree-in-bud appearance on the CT chest in the right lung was indicative of TB. However, neoplastic nodules could only be visualized on bronchoscopy. Although the coexistence of TB with malignancy may be a mere coincidence, numerous studies have been conducted that delineate the diverse potentialities of the pathogenesis underlying the co-occurrence of malignancy and TB. A study by Chan ED underlines the parallels and numerous potential molecular connections between MTB infection and carcinogenesis. According to some theories, immunosuppression brought on by cancer may make patients more vulnerable to opportunistic infections or the reactivation of dormant foci. Also, the risk of concurrent malignancy increases due to prolonged inflammation of chronic diseases, which leads to fibrosis and elastogenesis, further leading to the accumulation of carcinogens in the same area [10]. It is uncommon for this rare lung tumor and TB to coexist, as such a combination has not yet been documented in the literature. In our case, the coexistence of these two diseases posed a diagnostic challenge for the treating physician, especially given the rarity of the neoplasm. While primary pulmonary MEC of low grade typically has a favorable prognosis following surgical removal, the presence of concurrent untreated TB could alter the outcome. Patients with lymph nodal metastases, involved surgical tumor margins, or high-grade histomorphology should undergo postoperative chemotherapy [8]. Low-grade tumors have a higher five-year survival rate (~95%) compared to high-grade tumors (~43%) [6].

## Conclusions

Primary pulmonary MEC is a rare malignant lung tumor, and the concurrent presence of TB can obscure its diagnosis. Our patient had a low-grade lesion, which often has a favorable prognosis following primary surgical resection. However, the prognosis might be adversely affected by the concomitant pulmonary TB. If left untreated or only partially treated, TB is among the most common infectious diseases leading to mortality. The molecular profiles of these rare malignancies may assist in both diagnosis and treatment. We assert that this particular case warrants attention owing to its infrequency, highlighting the need for increased awareness of this diagnostic possibility.
